# Supplementary figures and images for: Improving the availability of antihypertensive drugs in the India Hypertension Control Initiative, India, 2019–2020
Source: PLoS One. 2023 Dec 14;18(12):e0295338. doi: 10.1371/journal.pone.0295338 (PMC10721057; doi:10.1371/journal.pone.0295338)

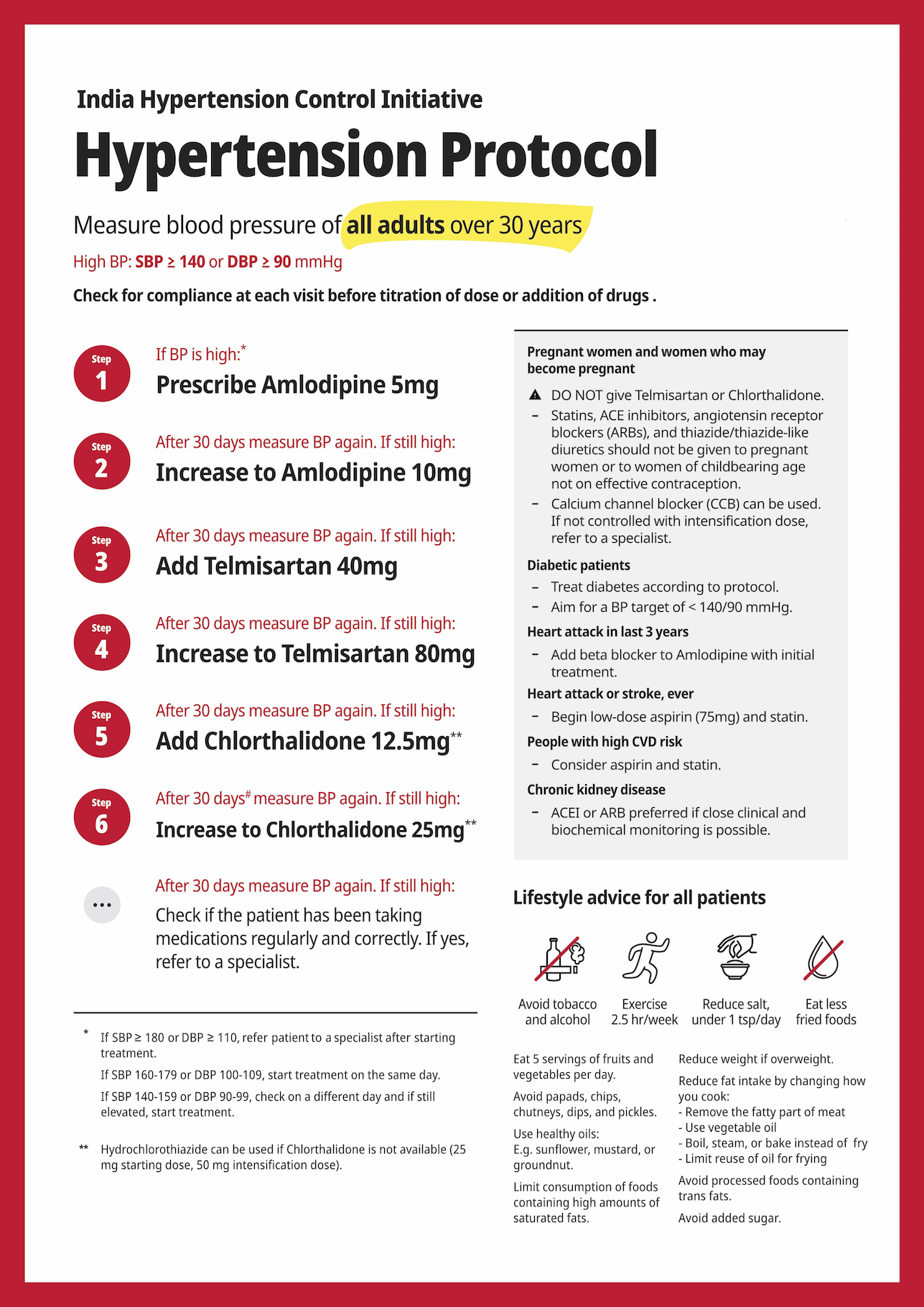

Supplement: S1 Fig — (TIFF) [file pone.0295338.s001.tiff]

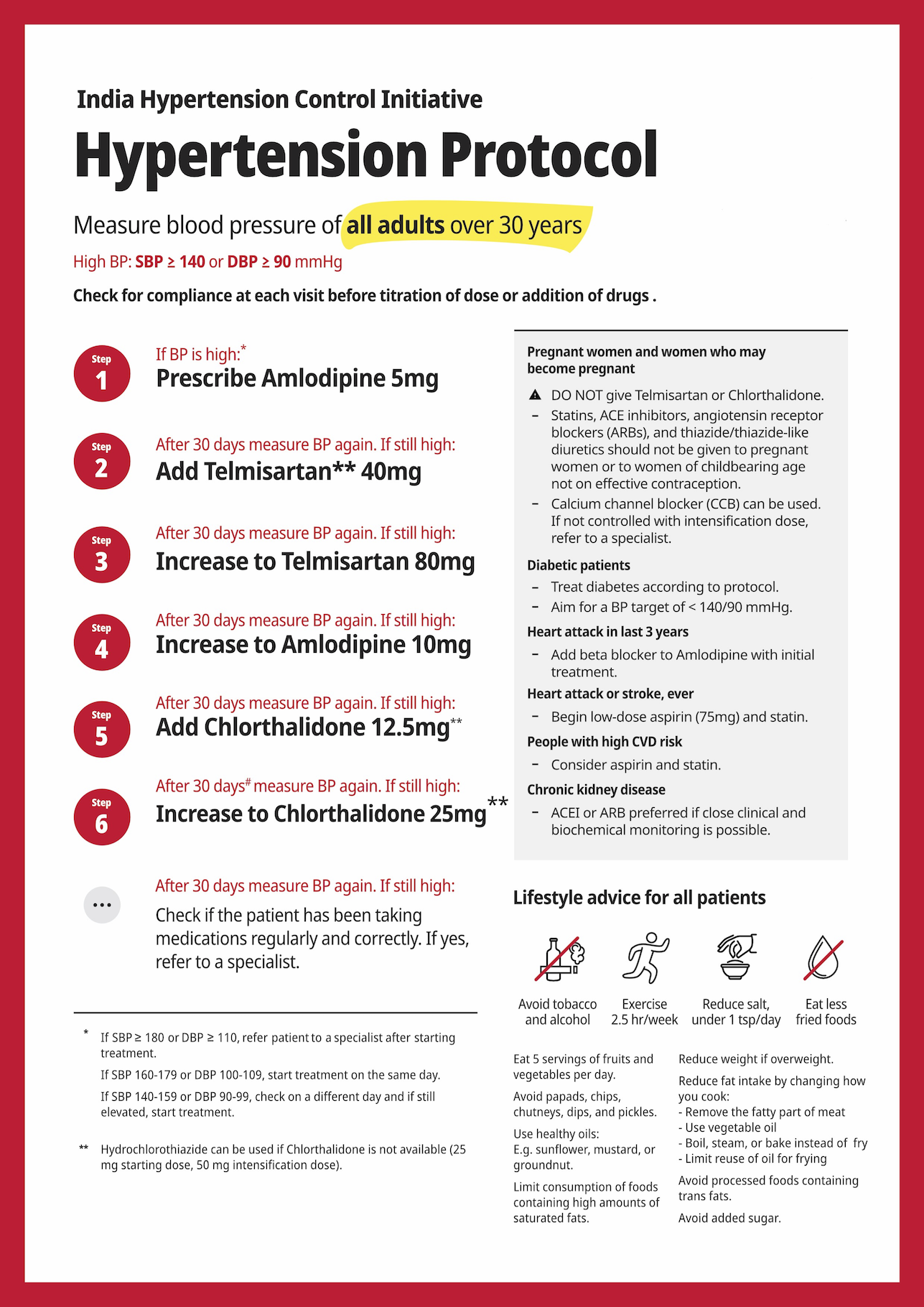

Supplement: S2 Fig — (TIFF) [file pone.0295338.s002.tiff]
