# Supplementary material for: Improving the availability of antihypertensive drugs in the India Hypertension Control Initiative, India, 2019–2020
Source: PLoS One. 2023 Dec 14;18(12):e0295338. doi: 10.1371/journal.pone.0295338 (PMC10721057; doi:10.1371/journal.pone.0295338)
Supplement: Attachment — Submitted filename: PONE-D-23-13904_reviewer_Aug 9.pdf [file pone.0295338.s009.pdf]

PLOS ONE

Improving the availability of antihypertensive drugs in the India Hypertension Control Initiative, India, 2019-2020

--Manuscript Draft--

**Manuscript Number:**	PONE-D-23-13904
**Article Type:**	Research Article
**Full Title:**	Improving the availability of antihypertensive drugs in the India Hypertension Control Initiative, India, 2019-2020
**Short Title:**	Availability of antihypertensive drugs in India
**Corresponding Author:**	Prabhdeep Kaur, DNB Medicine, MAE (Epidemiology)<br>ICMR-National Institute of Epidemiology<br>Chennai , Tamil Nadu INDIA
**Keywords:**	Hypertension, Antihypertensives, India, Procurement, Stock outs, Drugs
**Abstract:**	Background<br>Antihypertensive drug supply is sometimes inadequate in public sector health facilities in India. One of the core strategies of the India Hypertension Control Initiative (IHCI) is to ensure the availability of adequate drugs to manage hypertension in primary care facilities. We quantified the availability of antihypertensive drugs in 2019-20 and described the practices in supply chain management in 22 districts across four states of India.<br>Methods<br>Twenty-two districts from 4 states (Punjab, Madhya Pradesh, Telangana, and Maharashtra) were studied. We implemented interventions during 2018-19 to strengthen the supply chain at various levels under IHCI. We surveyed key stakeholders and described the practices and challenges in supply chain management. We collected data on drug procurement from 2018 to 2020 and drug availability from April 2019 to March 2020. Quantity procured, the proportion of facilities with stockout at the end of each quarter, and availability of drugs inpatient days were tabulated.<br>Results<br>All states selected drug- and dose-specific protocols with amlodipine as the initial drug and shifted to morbidity-based rather than historically-driven drug supply forecasting. The total number of tablets procured for all antihypertensive medications for the 22 districts in the four states increased from 16 million tablets in 2017-2018 to 160 million tablets in 2019-2020. The proportion of facilities with Amlodipine stock- out was below 5% in all states during the study period. Amlodipine stock was available for at least 60 patient days from the third quarter of 2019 onward in all districts.<br>Conclusions<br>This study demonstrates the feasibility of strengthening the procurement and supply chain management for antihypertensive drugs in a low-resource setting at scale. We recommend that best practices, including choosing a single protocol, basing supply on projected patient load rather than an increment from historical levels, use of simple, practical estimation and stock management tools, and meticulously managing drug stocks and resupply be replicated in other districts in India and in other LMIC to increase and sustain coverage of hypertension treatment.
**Order of Authors:**	Abhishek Kunwar, MBBS
	Prabhdeep Kaur, DNB Medicine, MAE (Epidemiology)
	Kiran Durgad
	Ganeshkumar Parasuraman
	Meenakshi Sharma
	Sudhir Gupta
	Balram Bhargava
**Additional Information:**	

Powered by Editorial Manager® and ProduXion Manager® from Aries Systems Corporation

Question	Response
**Financial Disclosure**<br><br>Enter a financial disclosure statement that describes the sources of funding for the work included in this submission. Review the submission guidelines for detailed requirements. View published research articles from *PLOS ONE* for specific examples.<br><br>This statement is required for submission and **will appear in the published article** if the submission is accepted. Please make sure it is accurate.<br><br>**Unfunded studies**<br>Enter: *The author(s) received no specific funding for this work.*<br><br>**Funded studies**<br>Enter a statement with the following details:<br>• Initials of the authors who received each award<br>• Grant numbers awarded to each author<br>• The full name of each funder<br>• URL of each funder website<br>• Did the sponsors or funders play any role in the study design, data collection and analysis, decision to publish, or preparation of the manuscript?<br>• **NO** - Include this sentence at the end of your statement: *The funders had no role in study design, data collection and analysis, decision to publish, or preparation of the manuscript.*<br>• **YES** - Specify the role(s) played.<br><br>* typeset	PK received grant from ICMR, New Delhi.
**Competing Interests**<br><br>Use the instructions below to enter a competing interest statement for this submission. On behalf of all authors, disclose any competing interests that could be perceived to bias this work—acknowledging all financial support and any other relevant financial or non-financial competing interests.	NO authors have competing interests

This statement is required for submission and **will appear in the published article** if the submission is accepted. Please make sure it is accurate and that any funding sources listed in your Funding Information later in the submission form are also declared in your Financial Disclosure statement.<br><br>View published research articles from *PLOS ONE* for specific examples.<br><br>**NO authors have competing interests**<br><br>Enter: *The authors have declared that no competing interests exist.*<br><br>**Authors with competing interests**<br><br>Enter competing interest details beginning with this statement:<br><br>*I have read the journal's policy and the authors of this manuscript have the following competing interests: [insert competing interests here]*<br><br>* typeset	
**Ethics Statement**<br><br>Enter an ethics statement for this submission. This statement is required if the study involved:<br><br>• Human participants<br>• Human specimens or tissue<br>• Vertebrate animals or cephalopods<br>• Vertebrate embryos or tissues<br>• Field research<br><br>Write "N/A" if the submission does not require an ethics statement.<br><br>General guidance is provided below. Consult the submission guidelines for detailed instructions. **Make sure that all information entered here is included in the Methods section of the manuscript.**	The study (ID no - NIE/IHEC/201709-02 is approved by ICMR-National Institution of Epidemiology, Chennai. Deidentified data and records review was used for analysis.

Powered by Editorial Manager® and ProduXion Manager® from Aries Systems Corporation

**Format for specific study types**<br><br>**Human Subject Research (involving human participants and/or tissue)**<br>• Give the name of the institutional review board or ethics committee that approved the study<br>• Include the approval number and/or a statement indicating approval of this research<br>• Indicate the form of consent obtained (written/oral) or the reason that consent was not obtained (e.g. the data were analyzed anonymously)<br><br>**Animal Research (involving vertebrate animals, embryos or tissues)**<br>• Provide the name of the Institutional Animal Care and Use Committee (IACUC) or other relevant ethics board that reviewed the study protocol, and indicate whether they approved this research or granted a formal waiver of ethical approval<br>• Include an approval number if one was obtained<br>• If the study involved *non-human primates*, add *additional details* about animal welfare and steps taken to ameliorate suffering<br>• If anesthesia, euthanasia, or any kind of animal sacrifice is part of the study, include briefly which substances and/or methods were applied<br><br>**Field Research**<br><br>Include the following details if this study involves the collection of plant, animal, or other materials from a natural setting:<br>• Field permit number<br>• Name of the institution or relevant body that granted permission	
**Data Availability**<br><br>Authors are required to make all data underlying the findings described fully available, without restriction, and from the time of publication. PLOS allows rare exceptions to address legal and ethical concerns. See the PLOS Data Policy and FAQ for detailed information.	Yes - all data are fully available without restriction

Powered by Editorial Manager® and ProduXion Manager® from Aries Systems Corporation

A Data Availability Statement describing where the data can be found is required at submission. Your answers to this question constitute the Data Availability Statement and **will be published in the article**, if accepted.<br><br>**Important:** Stating 'data available on request from the author' is not sufficient. If your data are only available upon request, select 'No' for the first question and explain your exceptional situation in the text box.<br><br>Do the authors confirm that all data underlying the findings described in their manuscript are fully available without restriction?	
**Describe where the data may be found in full sentences. If you are copying our sample text, replace any instances of XXX with the appropriate details.**<br><br>• If the data are **held or will be held in a public repository**, include URLs, accession numbers or DOIs. If this information will only be available after acceptance, indicate this by ticking the box below. For example: *All XXX files are available from the XXX database (accession number(s) XXX, XXX.*).<br>• If the data are all contained **within the manuscript and/or Supporting Information files**, enter the following: *All relevant data are within the manuscript and its Supporting Information files.*<br>• If neither of these applies but you are able to provide **details of access elsewhere**, with or without limitations, please do so. For example:<br><br>*Data cannot be shared publicly because of [XXX]. Data are available from the XXX Institutional Data Access / Ethics Committee (contact via XXX) for researchers who meet the criteria for access to confidential data.*<br><br>*The data underlying the results presented in the study are available*	https://data.mendeley.com/ under the title "Availability of antihypertensive drugs in project districts - India Hypertension Control<br>DOI: 10.17632/xs8dpz2z6v.1

from (include the name of the third party and contact information or URL).<br>• This text is appropriate if the data are owned by a third party and authors do not have permission to share the data.<br><br>* typeset	
Additional data availability information:	Tick here if the URLs/accession numbers/DOIs will be available only after acceptance of the manuscript for publication so that we can ensure their inclusion before publication.

Manuscript

Click here to access/download;Manuscript;Imroving drug availability_R1_21stMay2023 .docx

Title: Improving the availability of antihypertensive drugs in the India Hypertension Control Initiative, India, 2019-2020

Authors:
Abhishek Kunwar[1], Prabhdeep Kaur[2], Kiran Durgad[1], Ganeshkumar Parasuraman[2], Meenakshi Sharma[3], Sudhir Gupta[4], Balram Bhargava [3] on behalf of "India Hypertension Control Initiative (IHCI) collaboration."

List of authors (alphabetical order) for ***"India Hypertension Control Initiative (IHCI) Collaboration****."*
Sampada D Bangar[5], Vishwajit Bharadwaj[6], Rupali Bharadwaj[7], Sailaja Bitragunta[1], Sreedhar Chintala[8], Tapas Chakma[9], Tejpalsinh A Chavan[10], Sunil Dar[11], Bidisha Das[12], RS Dhaliwal[3], Sandeep Singh Gill[13], Tanu Jain[4], Padmaja Jogewar[14], Chakshu Joshi[15], Abhishek Khanna[16], Suhas N Khedkar[17], Ashish Krishna[18], Navneet Kumar[19], Vijay Kumar[20], Madhavi M[21], Anupam K Pathni[18], Satyendra N Ponna[22], Sravan K Reddy[23], Swagata K Sahoo[18], Ashish Saxsena[24], Bhawna Sharma[18], Shweta Singh[16], Gurinder B Singh[13], Sunny Swarnkar[4], Jatin Thakkar[25], Fikru T Tullu[2], Mohammed Abdul Wassey[26], Amol B Wankhede[27]

SB, VB, RB, SB, SC, TC, TAC, SD, BD, RSD, SSG, TJ, PJ, CJ, AK, SNK, AK, NK, VK, MM, AKP, SNP, SKR, SKS, AS, BS, SS, GBS, SS, JT, FTT, MAW, ABW

[1] Division of Noncommunicable Diseases, ICMR-National Institute of Epidemiology, Chennai
[2] Dept of Noncommunicable Diseases, WHO Country Office for India, New Delhi
[3] Indian Council of Medical Research (ICMR), New Delhi
[4] Directorate General of Health Services, Ministry and Health, and Family Welfare, New Delhi
[5] ICMR-National AIDS Research Institute, Pune
[6] IHCI Project, District NCD Cell, Bhandara (Maharashtra), WHO-India.
[7] IHCI Project, District NCD Cell, Chhindwara (Madhya Pradesh), WHO-India.
[8] IHCI Project, District NCD Cell, Karimnagar (Telangana), WHO-India.
[9] ICMR-National Institute of Research in Tribal Health, Jabalpur
[10] IHCI Project, District NCD Cell, Sindudurg (Maharashtra), WHO-India.
[11] IHCI Project, District NCD Cell, Hoshiarpur (Punjab), WHO-India.
[12] IHCI Project, District NCD Cell, Bhatinda (Punjab), WHO-India.
[13] State NCD Cell, Department of Health and Family Welfare, Government of Punjab, Chandigarh
[14] State NCD Cell, Directorate of Health Services, Government of Maharashtra, Mumbai
[15] IHCI Project, District NCD Cell, Ratlam (Madhya Pradesh), WHO-India.
[16] NPCDCS Cell, DGHS, New Delhi, WHO- India
[17] IHCI Project, District NCD Cell, Satara (Maharashtra), WHO-India.
[18] Resolve to Save Lives, New Delhi
[19] IHCI Project, State NCD Cell, Chandigarh (Punjab), WHO-India
[20] IHCI Project, District NCD Cell, Gurdaspur (Punjab), WHO-India.
[21] State NCD Cell, Department of Health, Medical and Family Welfare, Government of Telangana, Hyderabad
[22] IHCI Project, District NCD Cell, Warangal (Telangana), WHO-India.
[23] IHCI Project, State NCD Cell, Hyderabad (Telangana), WHO-India.
[24] State NCD Cell, Directorate of Health Services, Government of Madhya Pradesh, Bhopal
[25] IHCI Project, State NCD Cell, Bhopal (Madhya Pradesh), WHO-India.
[26] IHCI Project, District NCD Cell, Mahabubnagar (Telangana), WHO-India.
[27] IHCI Project, State NCD Cell, Mumbai (Maharashtra), WHO-India.

Corresponding author information:
Dr. Prabhdeep Kaur
Scientist F, ICMR- National Institute of Epidemiology,
Ayapakkam, Chennai – 600077
Email: kprabhdeep@nieicmr.org.in
Phone : +91-26136233

Word count: 4392
Figures: 4
Tables: 2

Abstract

Background

Antihypertensive drug supply is sometimes inadequate in public sector health facilities in India. One of the core strategies of the India Hypertension Control Initiative (IHCI) is to ensure the availability of adequate drugs to manage hypertension in primary care facilities. We quantified the availability of antihypertensive drugs in 2019-20 and described the practices in supply chain management in 22 districts across four states of India.

Methods

Twenty-two districts from 4 states (Punjab, Madhya Pradesh, Telangana, and Maharashtra) were studied. We implemented interventions during 2018-19 to strengthen the supply chain at various levels under IHCI. We surveyed key stakeholders and described the practices and challenges in supply chain management. We collected data on drug procurement from 2018 to 2020 and drug availability from April 2019 to March 2020. Quantity procured, the proportion of facilities with stockout at the end of each quarter, and availability of drugs inpatient days were tabulated.

Results

All states selected drug- and dose-specific protocols with amlodipine as the initial drug and shifted to morbidity-based rather than historically-driven drug supply forecasting. The total number of tablets procured for all antihypertensive medications for the 22 districts in the four states increased from 16 million tablets in 2017-2018 to 160 million tablets in 2019-2020. The proportion of facilities with Amlodipine stock- out was below 5% in all states during the study period. Amlodipine stock was available for at least 60 patient days from the third quarter of 2019 onward in all districts.

Conclusions

This study demonstrates the feasibility of strengthening the procurement and supply chain management for antihypertensive drugs in a low-resource setting at scale. We recommend that best practices, including choosing a single protocol, basing supply on projected patient load rather than an increment from historical levels, use of simple, practical estimation and stock management tools, and meticulously managing drug stocks and resupply be replicated in other districts in India and in other LMIC to increase and sustain coverage of hypertension treatment.

1. Introduction

In India, there are estimated to be more than 200 million adults living with hypertension [1]. However, less than half of them are aware of their hypertension status, and less than one-tenth of all people with hypertension have their blood pressure under control [2][3]. Most low- and low-middle-income countries have insufficient availability of antihypertensive medications[4]. Non-availability of blood pressure-lowering medications is strongly associated with poor control of hypertension [5]. In India, in a survey conducted across six states in 2004-05, the median availability of 27 essential medicines in the public sector ranged from 0–30 % [6]. A study conducted in public health facilities of two North Indian states found that the availability of antihypertensive drugs was only 60% [7[illegible] A national-level survey reported inadequate drugs for noncommunicable diseases in primary care facilities [8].

The India Hypertension Control Initiative (IHCI), launched in November 2017, aims to reduce premature cardiovascular deaths by strengthening hypertension management and control at primary and secondary public health care facilities. The initiative includes a standardized approach drawn from the World Health Organization (WHO) HEARTS technical package for cardiovascular disease management in primary health care [9]. Adopting standard, drug- and dose-specific hypertension treatment protocols and ensuring an uninterrupted supply of the selected protocol medications are two of the critical components of this initiative. The project was implemented in the public sector health facilities of 26 districts in five states from 2018-19. The project team worked closely with the state governments to improve the availability of antihypertensive drugs at the district and facility levels.

Improving access to essential medicines requires appropriate selection, financing, affordable prices, and supply systems.[10] Several interventions were initiated to improve the selection, procurement, and availability of antihypertensive drugs in the public sector health facilities implementing IHCI in 2018-19. We monitored the availability of drugs in these facilities in 2019-20 and worked with stakeholders to understand the progress and challenges in improving the availability of the protocol drugs. This paper describes practices in supply chain management, the number of drugs procured and drug availability at the facility level in the four Indian states implementing the IHCI project.

2. Methods

2.1. Design and setting

The study included all 22 districts from 4 states (Punjab, Madhya Pradesh, Telangana, and Maharashtra) implementing the IHCI. We conducted a cross-sectional survey of various stakeholders in four states between January and March 2020. The stakeholders included key officials from state-level procurement agencies and regional and district warehouses in the project districts. We considered only public sector facilities.

We collected data regarding drug procurement from 2018 to 2020 and drug availability at district and health facilities from April 2019 to March 2020 in the 22 districts. We estimated the availability of antihypertensive drugs in the standard treatment protocol at the health facility and district level, post-intervention in 2019-20, in 22 districts across four states of India.

2.2 Description of interventions

The project has five core strategies: protocol-based care, availability of adequate drugs, task sharing, patient-centered care, and a cohort-based monitoring system. Under IHCI, particular focus is rendered to ensure uninterrupted availability of drugs at all service delivery points. The project aimed to ensure timely purchase of adequate quantities of hypertension drugs, equitable

distribution of hypertension protocol drugs to service delivery points, and strengthening systems for monitoring and timely action. The project team developed multiple easy-to-adopt tools, curated interventions for improving supply chain efficiency, and extended continuous technical support. The critical interventions implemented from 2018 onwards to streamline the supply chain of antihypertensives were as follows:

Selection of drugs: We focused on procuring and supplying a limited number of antihypertensive drugs in line with the adopted treatment protocol (S1 Fig, S2 Fig). This approach helped streamline forecasting, efficient procurement with affordable drug costs, and improved stock keeping and distribution.

Forecasting drug requirements and budgeting: Before the IHCI, the traditional method of forecasting drug requirements was to project future consumption based on a proportion increase in past consumption. This approach is not suitable for scaling up programs. The morbidity-based method, by contrast, accounts for future program growth, but this method was not practiced before the IHCI due to the complexity of calculations required and lack of training. We developed a customized, easy-to-use forecasting tool (S3 File, S4 File). Based on the forecasted drug requirement, assistance was extended for budget planning and prioritizing through rationalization of existing funds and mobilization of additional funding through the national program and/or state funds [11].

Procurement: One of the most critical interventions is timely tendering prior to the expiry of the existing rate contract to ensure continuity of rate contract availability. We recommended a multi-year rate contract instead of annual tendering to reduce the administrative processes and bottlenecks. We also suggested multiple supplier empanelment to ensure the availability of an alternative supply source in case of failure/non-compliance by any one supplier. Many states

issued the procurement order for the entire year; however, there were challenges for the supplier and storage at the warehouse level. One of the more pragmatic strategies was to purchase orders with scheduled supply or scheduled procurement, which helped maintain adequate drug availability with minimal inventory holding. This approach helped mitigate the storage space challenge and minimized the risk of drug expiry and product deterioration during storage. The scheduled supply approach also acted as assurance for the supplier and proved helpful in planning and executing orders in time. Although most of the procurement was done at the state level, districts were provided funds and empowered to stop-gap local procurement if there was a disruption in the procurement/supply of drugs through an established state mechanism.

Storage and distribution: Every facility was advised to maintain 2-3 months' stock based on the total number of registered patients. Ready reckoners were developed to quickly assess stock position for patient load-linked indent/drug distribution and periodic (monthly) refilling (S5 File, S6 File)). The health facility or district store arranged vehicles to deliver drugs to service delivery points from the issuing stores.

Dispensing and monitoring: All states issued advisories to the districts to dispense at least 30 days of drugs per treated patient and monitor the stocks. We used easy-to-use and straightforward formats/tools for pharmacists and health care providers for inventory recording and reporting (S7 File). Health facilities with suboptimal stock levels were monitored every month at the district, state, and national levels with a feedback mechanism to drive prompt supply chain action to fill in the gaps.

Training and capacity building: We trained all pharmacists and program managers on the treatment protocol, annual requirement forecasting, monthly/periodic indent/stock refill based on the patients under treatment, stock level monitoring, and inventory records.

2.3 Data Collection

Review of documents:

We reviewed essential drug lists (EDLs), tender documents, procurement policy, past procurement data, drug distribution guidelines, and other relevant documents in the state-level procurement agencies, state NCD program unit, and district warehouses. The documents provided information regarding existing forecasting, procurement, and distribution practices.

Field-based observations:

The IHCI intervention team of public health specialists supported the state and district-level NCD program units. The team observed the practices regarding existing supply chain processes from procurement to dispensing to identify the challenges and to support streamlining the process.

Quantitative data for drugs:

We collected data on the annual procurement of all antihypertensive drugs for three financial years, i.e., 2017-18, 2018-19, and 2019-20, from state and district records.

We also collected data on hypertension protocol drugs stock throughout the study from all public sector health facilities of these districts at the end of each quarter [from quarter 2 (Q2) 2019 – quarter 1 (Q1) 2020] from stock ledgers and logistics management information system (LMIS) software. The quarterly drug stock reporting also consisted of a cumulative number of patients registered at the health facility under the IHCI to estimate the drug stock inpatient days. These data were extracted from the Simple digital hypertension management app in

Maharashtra and Punjab and from IHCI paper-based registers in Madhya Pradesh and Telangana.

2.4 Data analysis

We used the "Logistics cycle model" to identify the thematic areas for analysis.[12] We summarised various aspects of the supply chain, from selecting antihypertensive drugs to dispensing the drugs at the facility level.

a. *Qualitative analysis*

We analyzed and summarized policy documents, procurement records, monthly/annual reports, and in-depth interviews with stakeholders in five thematic areas (Fig 1).

Fig 1. Steps for antihypertensive medication supply chain management in the India Hypertension Control Initiative

b. *Quantitative analysis*

We analyzed three indicators using state, district, and health facility level data to assess if the interventions improved the availability of the antihypertensive drugs.

i. Quantity of antihypertensive drugs procured for three financial years (2017-18, 2018-19, and 2019-20)

ii. The proportion of health facilities with drug stock out Out of all implementing facilities, the number of facilities had nil stock of any protocol drug at the end of each quarter from Q2, 2019 to Q1, 2020.

iii. Drug stock in patient-days at the end of each quarter at the district level from Q2, 2019 to Q1, 2020.

'Drug stock in patient-days is the unit to measure how many days the available stock of a drug would last considering the number of patients under treatment and the average daily requirement of the drug per patient. The stock in patient-days for a drug is computed as 'Stock

available in a number of tablets' / (Total number of patients x Number of tablets required per day).

Under IHCI, each state adopted a drug and dose-specific stepwise hypertension treatment protocol. The program adopted initial assumptions on the proportion of patients started on treatment who are controlled at each step of the protocol. Based on the treatment protocol adopted and the assumed proportion of patients reaching blood pressure control at each treatment protocol step, the approximate requirement of each protocol drug per patient per month was calculated for two types of protocols (S5 File).

2.5 Human Subjects Protection

The ICMR-National Institute of Epidemiology, Chennai, institutional ethics committee approved the project. The study did not include any patient-level data. We collected the data and reviewed documents following the required administrative approval process.

3. Results

3.1 Selection of drugs:

Public procurement is done for drugs listed in the state’s EDL. Within each therapeutic category, several drugs are listed and classified by the health facility's level (primary, secondary, or tertiary) where the drugs are to be made available. All four states had developed hypertension treatment protocols as one of the strategies under the IHCI (Table 1).[13] The states had included the protocol drugs in the state-specific EDL. Before IHCI, antihypertensive drugs were mostly procured for secondary and tertiary health facilities. After IHCI implementation, all protocol drugs were included in the EDL for primary care facilities and secondary and tertiary care facilities.

Table 1. Drug selection, forecasting, procurement, storage and distribution practices for antihypertensive drugs in four Indian states, 2020.

Components	Maharashtra	Punjab	Madhya Pradesh	Telangana
**Selection of drugs**				
Availability of Essential Drug List	Yes	Yes	Yes	Yes
Protocol drugs in essential drugs list	Amlodipine, Telmisartan, Chlorthalidone	Amlodipine, Telmisartan, Chlorthalidone	Amlodipine, Telmisartan, Hydrochlorothiazide	Amlodipine, Telmisartan, Hydrochlorothiazide
Availability of protocol drugs at various health facility levels	All levels	All levels	All levels	All levels
**Forecasting**				
Frequency of annual demand generation	Yearly	Yearly	Yearly	Yearly
Method of drug forecasting	IHCI tool – Morbidity method	IHCI tool – Morbidity method	IHCI tool – Morbidity method	IHCI tool – Morbidity method
Review of indents	Yes	Yes	Yes	Yes
**Procurement**				
Name of nodal agency	Haffkine Bio-Pharmaceuticals Corp.Ltd.	Punjab Health Systems Corporation	Madhya Pradesh Public health Service Corporation Limited	Telangana State Medical Services & Infrastructure Development Corporation
Rate contract period	Quantity contract	2 years	2 years	2 years
Purchase order system	Centralized	Centralized	Decentralized	Centralized

Frequency of purchase order	Annual	Quarterly	Quarterly	Annual
Supply period	45 days	40 days	45 days	75 days
**Storage and distribution**				
Primary stores	District stores	Regional stores	District stores	Regional stores
Responsible for transportation from primary stores	Health facilities	Health facilities	Health facilities	Health facilities
Frequency of stock replenishment at health facilities	Monthly	Monthly	Monthly	Monthly

3.2 Forecasting drug requirements

Requirement forecasting is a crucial step in the supply chain to ensure the availability of adequate drugs. In all four states, essential drug requirements were collected from the district level annually for procurement and budget planning. The facilities submit the requirement to the district, and a consolidated report is submitted to the state. The annual requirements of antihypertensive drugs were estimated based on last year's consumption until 2017-18. The project team worked with the state-level stakeholders to estimate the requirement of the drug-using morbidity-based forecasting in 2018-19 and 2019-20 (Table 1). To forecast the annual drug requirement based on the total number of patients under care, expected new enrolment over the year, and the adopted treatment protocol, all 22 districts started using the forecasting tool developed for the project (S3 File, S4 File).

3.3 Procurement

All four states had fully functional state-owned procurement agencies (Table 1). The drug rate contract was renewed every two years in all states except Maharashtra, where a quantity contract was used. The drug purchase order system was centralized in all states except Madhya Pradesh. In Madhya Pradesh, districts were authorized to purchase from manufacturers directly. Annual purchase orders were placed in Maharashtra and Telangana, whereas quarterly in Madhya Pradesh and Punjab.

All state procurement agencies procured drugs directly from the manufacturers. Good manufacturing practices compliance certificate and production experience of at least three years were mandatory to procure the drugs from credible sources. Although the manufacturers mandatorily do quality control tests before supplying stocks to the states, all state procuring agencies retest the samples at independent empanelled testing laboratories to confirm the quality of the drug before the batches are released to the health facilities.

IHCI field teams observed the key challenges which led to delays in procurement were non-availability of rate contracts, lack of multiple suppliers, delays in tendering and placing purchase orders, delayed or partial supply by vendors, delays in receiving funds and lack of well-defined budget heads for antihypertensive drugs. As per procurement data, the procurement improved in 2019-20, but several challenges could not be addressed. The effective interventions at the procurement level were close monitoring and follow-up of tenders by state NCD nodal units, providing reliable estimates by forecasting, the collaboration between procurement cell and NCD unit for purchase scheduling, accelerating the quality control process to reduce quarantine time, and strategic procurement when contract nears expiry. At the district level, local procurements helped fill the gaps when the supply from the state was delayed.

All states increased the quantities of antihypertensive drugs procured for the project districts after the project's initiation (Fig 2). In Madhya Pradesh, there was a three-fold increase in the procurement of amlodipine and telmisartan in 2018-19 and a six-fold increase in the procurement of diuretics. The procurement of amlodipine in 2019-20 was 3-10-fold higher in 2019-20 compared to 2017-18 in Telangana, Maharashtra and Punjab. Punjab and Maharashtra did not procure telmisartan in 2017-18. Telmisartan was procured in small quantities in Punjab in 2018-19, and nearly 4 million tablets were procured in 2019-20. The procurement of telmisartan in Telangana was lower in 2018-19 compared to the previous year due to delays in supply from the manufacturers. In Maharashtra, delays in the tender process led to lower procurement in 2019-20 compared to the previous year. Except for Madhya Pradesh, the procurement of diuretics was not done in 2017-18 and only after the inclusion of chlorthalidone or hydrochlorothiazide was initiated in other states in the subsequent years. The total number of tablets procured of all antihypertensive medications for the 22 districts in the

four states increased from 16 million tablets in 2017-2018 to 160 million tablets in 2019-2020 (Fig 3).

Fig 2. Volume of procurement of antihypertensive drugs (in millions of tablets) by type of drug and by state for 2017-18, 2018-19, 2019-20.

Fig 3. Volume of procurement of all antihypertensive drugs (in millions of tablets) drugs overall and by type of drug for all study districts for 2017-18, 2018-19, 2019-20.

3.4 Storage and distribution

Drugs are directly delivered to the district stores based on the purchase orders in Maharashtra and Madhya Pradesh. The district stores catered to the health facilities. In Punjab and Telangana, regional drug warehouses received the drugs from the suppliers and further distributed them to the district stores, which supplied all health facilities in the district.

At the facility level, stocks were replenished monthly (Table 1). The refill quantity was assessed using ready reckoners (S5 File, S6 File). The monthly cycle helped in overcoming the storage-related challenges at the facility level. The pharmacists from health facilities sent an indent, arranged transport, and collected drugs from the primary/district levels stores, usually once a month. The distribution of drugs to the health facilities is based on a requisition or pull system, and the quantity to be issued is based on requests placed by the health facilities. Current practices utilise ambulances and other program monitoring vehicles available at health facilities to pick up the drug stocks. Thus, the availability of drugs is affected by the availability of the vehicle and can lead to a shortage and stockout of drugs at health facilities even when there are adequate stocks at the district/issuing stores. Given these constraints, district-level warehouses was easier to access than regional or state-level warehouses.

IHCI field teams observed three key challenges. The first was the availability of transport for collecting drugs from the district warehouses. The second challenge was a lack of

understanding among pharmacists regarding estimating drug requirements for requisitions sent to the drugs stores. The third challenge was the lack of rational distribution of drugs which led to excess stocks in some facilities and stockouts in others. Under IHCI, min-max inventory levels were affixed for the health facilities. A ready reckoner was prepared for pharmacists to assess the antihypertensive drugs required for a given number of patients enrolled in the treatment (S6. File). The pharmacists reported that the ready reckoner helped prepare monthly requisition and reduced stockouts.

3.5 Availability of drugs at the district level and point of care

The project began in 2018 and enrolled 68,982 number of patients in the first year and 283,474 in 2019. A total number of patients enrolled under IHCI in the 22 districts increased from 83,553 in Jan 2019 to 441,929 in March 2020.

Amlodipine stock was available for at least 60 patient-days in all districts at the end of every quarter from Q2, 2019 in Punjab, Maharashtra and Madhya Pradesh and August 2019 onwards in Telangana (Fig 4). Telmisartan/Losartan was available for 60 patient days or higher for all nine months in Punjab and Maharashtra. The availability was below 60 patient days for two months (Jan 2019 and March 2019) in Maharashtra and four months (Dec 2019 to March 2020) in Telangana. Diuretics were supplied from August 2019 onwards in Telangana and were available for 120 patient days or higher after that. In Maharashtra and Madhya Pradesh, diuretics were available for 100 patient days or higher throughout nine months (Fig 4).

Fig 4. Antihypertensive drug availability inpatient days by the state for four states of India, Jun 2019 – March 2020.

The proportion of facilities with amlodipine stock out was below 5% in all states during the study period (Table 2). Less than 10% of facilities had telmisartan/losartan stockouts between June 2019 and Dec 2020. In March 2020, Punjab had 10% stockouts and Telangana 12%

stockouts for telmisartan/losartan. Stockouts were higher for diuretics compared to the other two drugs.

Table 2 Proportion of facilities with stockouts (no drugs available) at the end of each quarter between June 2019 – March 2020.

	June 2019			September 2019			December 2019			March 2020		
	Total No. of health facilities	No. of Facilities with stock out	% with stock out	Total No. of health facilities	No. of Facilities with stock out	% with stock out	Total No. of health facilities	No. of Facilities with stock out	% with stock out	Total No. of health facilities	No. of Facilities with stock out	% with stock out
**Amlodipine**												
Punjab	155	3	1.9	155	9	5.8	155	13	8.4	155	13	8.4
Madhya Pradesh	155	0	0.0	171	0	0.0	171	1	0.6	171	0	0.0
Maharashtra	218	2	0.9	218	3	1.4	218	8	3.7	218	3	1.4
Telangana	199	2	1.0	199	3	1.5	201	1	0.5	201	0	0.0
**Telmisartan**												
Punjab	155	11	7.1	155	24	15.5	155	21	13.5	155	33	21.3
Madhya Pradesh	155	7	4.5	171	4	2.3	171	5	2.9	171	1	0.6
Maharashtra	218	5	2.3	218	4	1.8	218	13	6.0	218	23	10.6
Telangana	199	9	4.5	199	4	2.0	201	11	5.5	201	25	12.4
**Diuretic**												
Punjab	155	106	68.4	155	93	60.0	155	117	75.5	155	155	100.0
Madhya Pradesh	155	66	42.6	171	17	9.9	171	10	5.8	171	19	11.1
Maharashtra	218	7	3.2	218	21	9.6	218	30	13.8	218	47	21.6
Telangana	199	199	100.0	199	65	32.7	201	11	5.5	201	14	7.0

3.6 Dispensing to patient

Prior to IHCI project implementation, the number of days for which drugs were dispensed varied from seven days to 30 days depending on the availability of drugs. After initiation of IHCI, all four states issued guidelines, and medical officers and pharmacists were trained for prescribing and dispensing the antihypertensive drugs for 30 days.

3.7 Financing and logistic management information systems

In India, all states procured drugs by pooling funds from the national level and state level [11]. The annual budget is prepared by the states and submitted to state and central government. All four states have digital logistic management information systems (LMIS) for managing drug procurement, inventory, distribution and monitoring. This system was used in Madhya Pradesh and Telangana since 2014 and Maharashtra and Punjab since 2017 at all levels starting from state level to primary health care level. The institutional mechanism was in place for monthly drug stock reviews in all four states. Regular monitoring and supervisory visits were done to all types of drug stores, and records were maintained with respective supervisors. All state pharmacists used digital platform reports for periodic review and appropriate action. However, none of the district officials used digital platform reports.

4. Discussion

We assessed the functioning of the steps in the antihypertensive drugs supply chain from selection, forecasting practices, and procurement to availability at the point of care delivery in 22 districts over the beginning phase of IHCI implementation (2018-2020). Overall, procurements of antihypertensive protocol drugs increased to volumes closer to patient-day needs for the growing program, and their availability was sufficient even at the primary health care level. States planning to scale up hypertension control programs should adopt a package of

interventions including drug-dose specific treatment protocol, forecasting using tools, multi-year rate contracts, a ready reckoner at the facility level and key indicators for monitoring drug availability. The interventions such as drug-dose specific treatment protocol, multi-year rate contracts and monthly monitoring of drug availability at the facility level are relevant for all disease conditions. However, forecasting tools and ready reckoners are useful for any chronic disease program where patients need to take drugs on a long-term basis.

WHO recommends rational selection of essential drugs as one of the critical strategies for improving access to treatment.[10] The IHCI project team worked with stakeholders to develop simple drug and dose-specific protocols [13]. The protocols were consistent with the recent WHO hypertension treatment guidelines [14]. Globally, many countries have developed drug-specific protocols for hypertension treatment [15]. Prior to implementing the IHCI, there was no uniformity in the prescription practices, making drug procurement challenging. The protocols are beneficial for program managers to plan procurement. Program managers need to procure fewer drugs, each in larger quantities, which pools demand and lead to an economy of scale, thereby reducing the unit cost of the drugs. The states procured only three drugs in the project districts for the primary care facilities, making the supply chain more efficient.

One of the significant challenges in the supply chain is the lack of accurate estimation of drug requirements at the facility and district levels. Procurement of insufficient quantities of medicines due to poor forecasting and unexpected demand challenges has been documented in the LMIC health systems [16]. Before implementing IHCI, the estimated requirements were mainly based on the previous year's consumption. The consumption-based approach underestimates the volume required if the facility has repeated stockouts or the program is expanding and treating more patients. Under the IHCI, hypertension protocol-based annual drug forecasting based on the existing patient load and additional expected patient load made

the estimates more reliable and reduced stockouts. Estimating periodic stock replenishment at health facilities requires sound information systems that track the number of patients on treatment rather than the number of visits to the health centre. Prior to implementing IHCI, only the aggregate data on the number of patient visits was collected and reported, but there was a lack of a registry. Once the information system was in place, ready reckoners based on patient registration helped pharmacists estimate monthly demand. The morbidity-based estimation method will be useful until the enrolment of a large proportion of patients who are expected to take treatment in the public sector. Once the district has achieved saturation based on utilization of public sector health facilities, the transition to the consumption-based method can be considered based on the assessment.

In the past decade, most Indian states have developed a centralized procurement mechanism under a dedicated procurement agency at the state level. To implement the interventions mentioned in this study, the states should have a department/agency that oversees the procurement process, a well-defined procurement policy to govern supplier selection and contract management, a coordination mechanism between the user department and procurement unit, a risk mitigation plan for supply/quality failures and timely payment mechanism. In the study districts, procurement improved with better planning, continuous follow-up with various stakeholders, and forecasting over the years. As measured by patient days, overall availability of the drugs was adequate in the study period, and there were few stockouts for the first two protocol drugs. A systematic review of studies from LMICs reported that procurement challenges included insufficient funds, delays in procurement, and insufficient procurement due to poor forecasting [16]. Robust governance, efficiency and transparency in procurement and contracting practices are essential to improve drug availability. We could not fully document and address these challenges in our study. We need

more in-depth studies to explore these challenges and better overall coordination between the program managers and procurement teams to reduce delays.

At least two protocol drugs were available throughout the study period. Several studies from India and other LMICs have documented poor availability of essential drugs.[7, 17-21] A national-level survey of public and private sector facilities reported poor NCD drugs and technologies in primary care. In the districts where the national NCD program was implemented, less than 1% of community health centres (which cater to approximately 100,000 population) and 17% of district hospitals were fully equipped with medicines and technologies to manage hypertension and cardiovascular diseases [8]. In Punjab, a survey of health facilities reported a 60% availability of antihypertensive drugs in 2015 [7]. Our data indicated an improvement in the availability of drugs in Punjab compared to 2015. Our experience reinforces the importance of building capacity and implementing good supply chain management practices to improve availability and reduce stockouts.

The limitation of our study was that we could not compare our data with the district-level availability of drugs before the start of the project. We used the procurement data as a surrogate for the availability of drugs in 2017-18 and 2018-19. We did nor design a separate study for supply chain interventions. In the initial planning and implementation phase, lack of drugs were a major challenge. Hence, we planned supply chain interventions described in the manuscript. Our experience can offer important insights to researchers and program managers working in low resource settings and can be used to design more structured well designed studies for supply chain interventions to improve availability of NCD drugs in context of LMIC.

We demonstrated the feasibility of strengthening procurement and supply chain management for antihypertensive drugs in a low-resource setting at scale. We were able to document improvement in several important areas, including the adoption of the treatment protocol, increased procurement volumes due to forecasting and collaboration among various stakeholders to match requirements with program scale-up, monthly stock monitoring at the facility level, improved availability of antihypertensive drugs included in the protocol in primary care facilities and minimum 30 days refills for the patients. There were challenges which could not be fully addressed, including timely tendering to ensure uninterrupted rate contracts, multiple suppliers as a risk mitigation strategy, timely placement of purchase ordering and supply schedules and timely availability of transport at the facility level for collecting drugs from the warehouse. Effective practices established in the IHCI can be replicated in other districts in India and in other LMICs to increase the coverage of hypertension treatment. The interventions to further strengthen the governance and adoption of best procurement practices should be sustained to ensure the availability of drugs for NCDs.

Acknowledgment

We thank the field-level health care workers, nurses, doctors, and district-level health officials for providing services for managing hypertension as part of the IHCI. We thank Senior Treatment Supervisors for their role in capacity building and supportive supervision in the project districts. We thank the ICMR Task Force Chair, Dr. Ambuj Roy, and all other experts for their valuable inputs in designing and implementing the project.

Supporting information

S1 Fig. Sample treatment protocol 1
S2 Fig. Sample treatment protocol 2
S3 File. Drugs forecasting tool for sample treatment protocol 1
S4 File. Drugs forecasting tool for sample treatment protocol 2
S5 File. Monthly drug requirement matrix for sample protocols 1 and 2
S6 File. Drug adequacy ready reckoner for sample treatment protocols 1 and 2

S7 File. Data collection format for the stock position of antihypertensive drugs inpatient days

References

1. Geldsetzer P, Manne-Goehler J, Theilmann M, Davies JI, Awasthi A, Vollmer S, et al. Diabetes and hypertension in India: a nationally representative study of 1.3 million adults. JAMA internal medicine. 2018;178(3):363-72.
2. Mathur P, Kulothungan V, Leburu S, Krishnan A, Chaturvedi HK, Salve HR, et al. National noncommunicable disease monitoring survey (NNMS) in India: Estimating risk factor prevalence in adult population. PloS one. 2021;16(3):e0246712.
3. ICMR-NCDIR. National Non-communicable Disease Monitoring Survey (NNMS) 2017–18.: ICMR-National Institute of Disease Informatics and Research, Bangaluru; 2021 [cited 2021 28th March]. Available from: https://ncdirindia.org/nnms/.
4. Husain MJ, Datta BK, Kostova D, Joseph KT, Asma S, Richter P, et al. Access to cardiovascular disease and hypertension medicines in developing countries: an analysis of essential medicine Lists, price, availability, and affordability. Journal of the American Heart Association. 2020;9(9):e015302.
5. Attaei MW, Khatib R, McKee M, Lear S, Dagenais G, Igumbor EU, et al. Availability and affordability of blood pressure-lowering medicines and the effect on blood pressure control in high-income, middle-income, and low-income countries: an analysis of the PURE study data. The Lancet Public Health. 2017;2(9):e411-e9.
6. Kotwani A, Ewen M, Dey D, Iyer S, Lakshmi P, Patel A, et al. Prices & availability of common medicines at six sites in India using a standard methodology. Indian journal of medical research. 2007;125(5):645.
7. Prinja S, Bahuguna P, Tripathy JP, Kumar R. Availability of medicines in public sector health facilities of two North Indian States. BMC Pharmacology and Toxicology. 2015;16(1):43. DOI: 10.1186/s40360-015-0043-8.
8. Krishnan A, Mathur P, Kulothungan V, Salve HR, Leburu S, Amarchand R, et al. Preparedness of primary and secondary health facilities in India to address major noncommunicable diseases: results of a National Non-communicable Disease Monitoring Survey (NNMS). BMC health services research. 2021;21(1):1-12.
9. World Health Organisation. Cardiovascular disease -HEARTS technical package Geneva: World Health Organisation,; 2017 [cited 2020 19th August]. Available from: https://www.who.int/cardiovascular_diseases/hearts/en/.
10. World Health Organisation. Equitable access to essential medicines: a framework for collective action. Geneva2004 [cited 2021 10th September]. Available from: http://whqlibdoc.who.int/hq/2004/WHO_EDM_2004.4.pdf.
11. Choudhury M, Mohanty RK. Role of National Health Mission in Health Spending of States: Achievements and Issues. New Delhi: National Institute of Public Finance and Policy, 2020.
12. John Snow Inc. The supply chain managers's handbook - A practical guide to the management of health commodities Arlington, VA: John Snow Inc,; 2017 [cited 2021 10th September]. Available from: https://www.jsi.com/resource/the-supply-chain-managers-handbook/.
13. ICMR-NIE. India Hypertension Control Initiative: State Hypertension Protocols: ICMR-NIE, Chennai; 2021 [cited 2021 16th Aug]. Available from: https://www.ihci.in/resources/protocols.
14. World Health Organisation. Guideline for the pharmacological treatment of hypertension in adults Geneva: World Health Organisation,; 2021 [cited 2021 11th November]. Available from: https://apps.who.int/iris/bitstream/handle/10665/344424/9789240033986-eng.pdf
15. DiPette DJ, Goughnour K, Zuniga E, Skeete J, Ridley E, Angell S, et al. Standardized treatment to improve hypertension control in primary health care: The HEARTS in the Americas Initiative. The Journal of Clinical Hypertension. 2020;22(12):2285-95.
16. Olaniran A, Briggs J, Pradhan A, Bogue E, Schreiber B, Dini HS, et al. Stockouts of Essential Medicines Among Community Health Workers (CHWs) In Low-And Middle-Income Countries (LMICs): A Systematic Literature Review of The Extent, Reasons, And Consequences. 2021.
17. van Mourik MSM, Cameron A, Ewen M, Laing RO. Availability, price and affordability of cardiovascular medicines: A comparison across 36 countries using WHO/HAI data. BMC Cardiovascular Disorders. 2010;10(1):25. DOI: 10.1186/1471-2261-10-25.

18. Cameron A, Ewen M, Ross-Degnan D, Ball D, Laing R. Medicine prices, availability, and affordability in 36 developing and middle-income countries: a secondary analysis. The Lancet. 2009;373(9659):240-9. DOI: https://doi.org/10.1016/S0140-6736(08)61762-6.
19. Mendis S, Fukino K, Cameron A, Laing R, Filipe Jr A, Khatib O, et al. The availability and affordability of selected essential medicines for chronic diseases in six low-and middle-income countries. Bulletin of the world health organization. 2007;85:279-88.
20. Wollum A, Gabert R, McMillan CR, Daly JM, Reddy P, Bhatt P, et al. Identifying gaps in the continuum of care for cardiovascular disease and diabetes in two communities in South Africa: Baseline findings from the HealthRise project. PLOS ONE. 2018;13(3):e0192603. DOI: 10.1371/journal.pone.0192603.
21. Satheesh G, Sharma A, Puthean S, Ansil TP M, E J, Raj Mishra S, et al. Availability, price and affordability of essential medicines for managing cardiovascular diseases and diabetes: A statewide survey in Kerala, India. Tropical Medicine & International Health. 2020;25(12):1467-79.

Figure 1

Click here to access/download;Figure;Fig 1.tiff

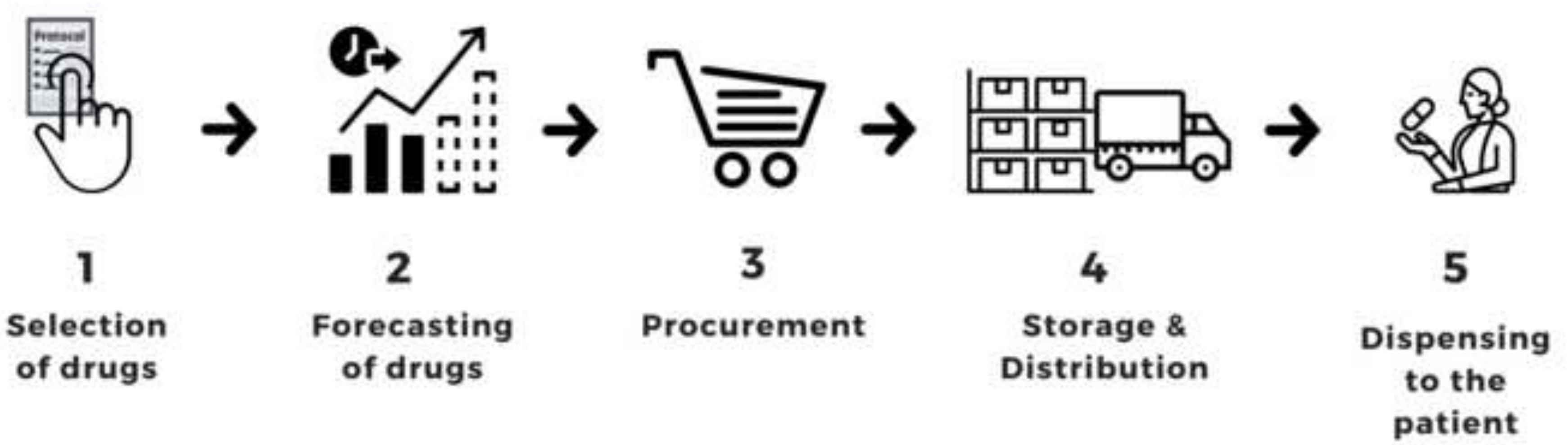

Figure 2

Click here to access/download;Figure;Fig 2.tiff

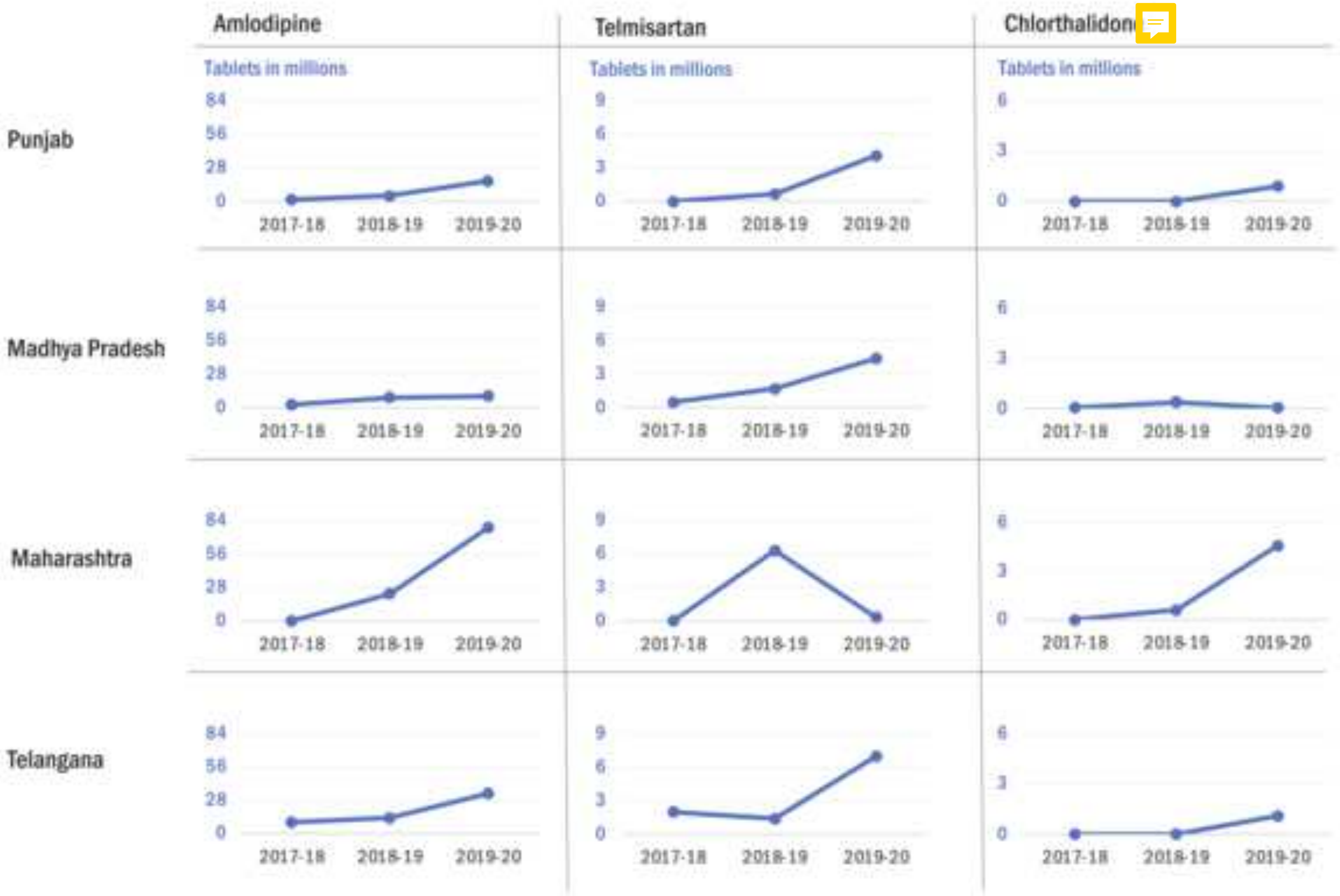

Figure 3

Click here to access/download;Figure;Fig 3.tiff

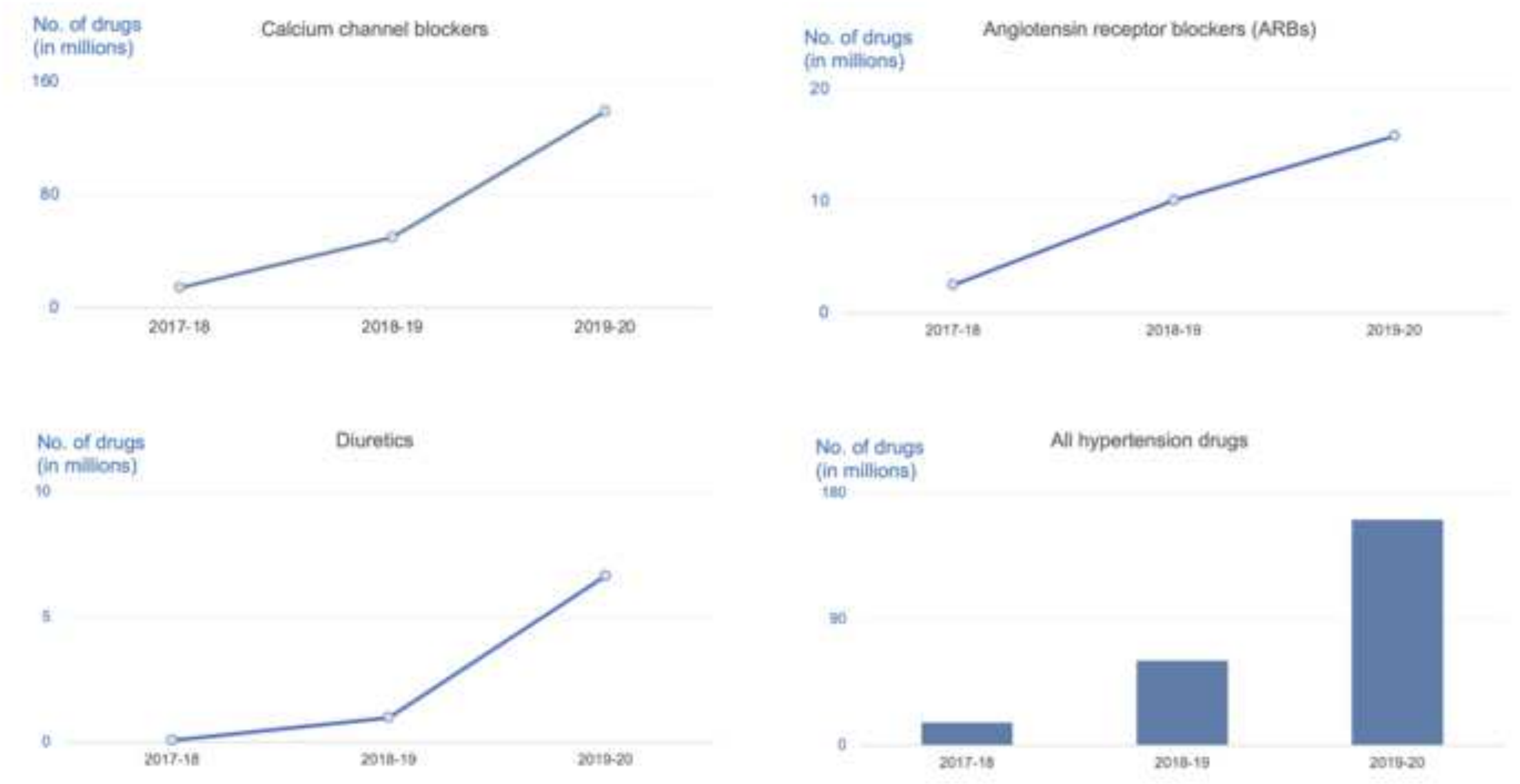

Figure 4

Click here to access/download;Figure;Fig 4.tiff

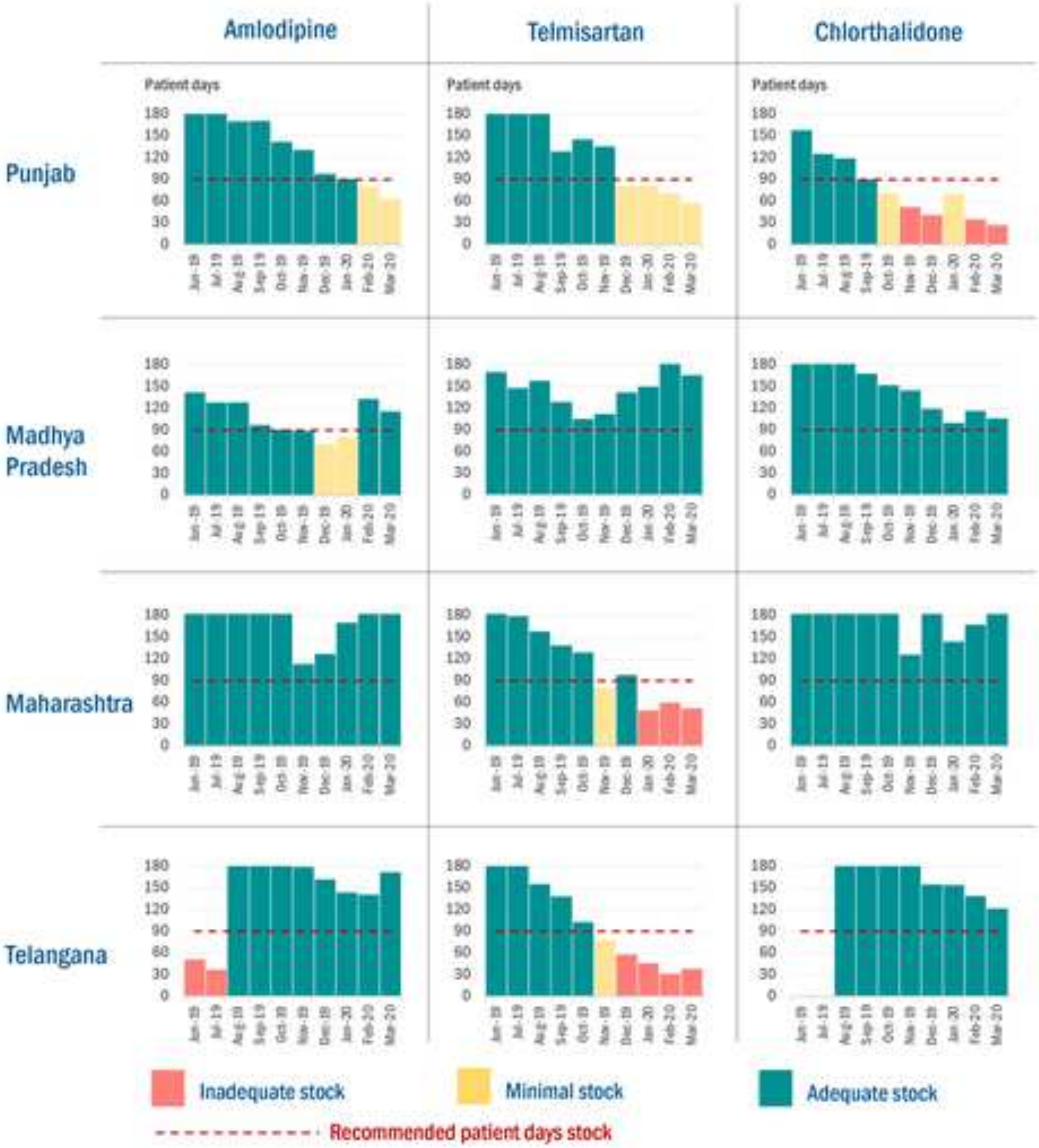

S1 Fig

Click here to access/download
Supporting Information
S1 Fig.tiff

S2 Fig

Click here to access/download
Supporting Information
S2 Fig.tiff

S3 File

Click here to access/download
Supporting Information
S3 File.xlsx

S4 File

Click here to access/download
Supporting Information
S4 File.xlsx

S5 File

Click here to access/download
Supporting Information
S5 File.xlsx

S6 File

Click here to access/download
Supporting Information
S6 File.xlsx

S7 File

Click here to access/download
Supporting Information
S7 File.xlsx

Improving the availability of antihypertensive drugs in the India Hypertension Control Initiative, India, 2019-2020

General Comment:

Author response:

We are thankful to the Reviewer for their positive comments and putting forward very relevant queries, addressing to which shall help making the paper more robust. Public health supply chains are complex with multiple stakeholders and multi-level decision making. The policies and process framework also differ from state to state. Public health systems in India have limited experience of handling large scale chronic disease control program which requires uninterrupted availability of specific drugs. Under IHCI we planned interventions for each step of the supply chain process, starting from the product selection to dispensing. While the intervention levels are generic, the implementation plan and tools were curated to suit requirements of a scaling-up program.

What evidence does it create about the impact of the interventions carried out and how they impact difference performance objectives. (e.g. stock out rates being one of them).

Uninterrupted availability of drugs is single most important indicator of supply chain system performance. While our study does not have the baseline data to present magnitude of improvement, the evidence is based on three quantitative indicators as described in the methods and results. The indicators include

i. Quantity of antihypertensive drugs procured for three financial years (2017-18, 2018-19, and 2019-20)

ii. The proportion of health facilities with drug stock out Out of all implementing facilities, the number of facilities had nil stock of any protocol drug at the end of each quarter from Q2, 2019 to Q1, 2020.

iii. Drug stock in patient-days at the end of each quarter at the district level from Q2, 2019 to Q1, 2020.

Page 9, section 2.4 (b)

Should other states carry out the full package of interventions described?

States planning to scale up hypertension control programs should adopt a package of interventions including drug-dose specific treatment protocol, forecasting using tools, multi-year rate contracts, a ready reckoner at the facility level and key indicators for monitoring drug availability.

Page 19, first para discussion

While I agree with the authors that choosing a single protocol, forecasting based on patient enrolment vs historical consumption, and basic stock management may be worth expanding to all districts, it is unclear if those interventions will work without good procurement and contracting practices, transparency, and robust governance etc. So how does the choice and prioritization depend on the system, governance, and context in a given state?

We appreciate the reviewer for highlighting the importance of procurement governance in strengthening the supply chain system. We have highlighted some of the issues on page XX. We acknowledge that study did not fully address these issues. This has been added to the discussion. "Robust governance, efficiency and transparency in procurement and contracting practices are essential to improve drug availability. We could not fully document and address these challenges in our study. We need more in-depth studies to explore these challenges and better overall coordination between the program managers and procurement teams to reduce delays."

Page 21, Para 2

Is there a pre-requisite set of conditions in procurement governance which have to be in place before this can be implemented in a state?

Yes, we agree with the reviewer. The government of India encourages all states to develop autonomous procurement agencies. To implement the interventions, the states should have a department/agency that oversees the procurement process, a well-defined procurement policy to govern supplier selection and contract management, a coordination mechanism between the user department and procurement unit, a risk mitigation plan for supply/quality failures and timely payment mechanism.

Page 21, para 2

Specific comments

Some of the supply chain processes of forecasting, procurement, etc are the same for all health products and are not unique to antihypertensives alone. So, would such an intervention work for any drug on the EML if the package of interventions is implemented? it will be useful to bring out more clearly what parts (if any) of the supply chain for antihypertensives are different from essential medicines more broadly?

Author response

Thanks for highlighting this important issue. The interventions such as drug-dose specific treatment protocol, multi-year rate contracts and key indicators for monitoring drug availability at the facility level are relevant for all disease conditions. However, forecasting tools and ready reckoners are useful for hypertension or other chronic diseases where patients need to take drugs on a long-term basis.

Page 20, para 1

There is literature that compares when to use morbidity base forecasts, patient enrolment-based forecasts, historical stock data-based forecasts, etc. A key answered question here is when should a system transition to using consumption-based forecasts? Initially as the program is ramping up it makes sense to use patient enrolment-based forecasts but a few years down as the program achieves equilibrium is there an inflection point where a different forecasting algorithm may be needed?

Author response

We thank the reviewer for this important query. Given the high prevalence and unmet needs, it is reasonable for the public sector to target the enrolment of at least 50 % of the total hypertensive patients. Hence morbidity-based methods are relevant until a large proportion of expected patients are enrolled. We have added this to the discussion. “The morbidity-based estimation method will be useful until the enrolment of a large proportion of patients who are expected to take treatment in the public sector. Once the district has achieved saturation based on utilization of public sector health facilities, the transition to the consumption-based method can be considered based on the assessment.”

Page 21

The paper does not delve into the frequency of replenishment of each health facility. More frequent replenishment/delivery to health facilities reduces the reliance on facility forecasts for long intervals.

Author response:

We thank the reviewer for the opportunity to clarify the long-term forecasting and periodic stock replenishment. Typically, drug requirement forecasting is done annually to prepare a procurement plan and budget. Therefore long-term forecasting is a pre-requisite for ensuring an adequate quantity of drugs can be procured in the year. We have now included the frequency of stock replenishment at health facilities in the results section (table 1, page 12). Details added in the results. "At the facility level, stocks were replenished on a monthly basis. The refill quantity was assessed using ready reckoners (S5 File, S6 File). The monthly cycle helped in overcoming the storage-related challenges at the facility level."

Page 15, section 3.4 para 2

Similarly, the paper does not delve into the supply chain network design aspects. Is it OK to have state level and district level stores? Should there be more direct distribution from State warehouses to health facilities?

We thank the reviewer for suggesting including supply chain network design aspects in the manuscript. Two of the four states included in the study had district-level stores, while Punjab and Telangana have regional stores from which the drugs are distributed to the health facilities (table 1). As mentioned earlier, the facilities do monthly refills. The pharmacists from health facilities sent an indent, arranged transport, and collected drugs from the primary/district levels stores, usually once a month. Due to transport challenges, it isn't easy to visit regional /state stores, which are far away from facilities. We have added this in the manuscript "Given these constraints, district-level warehouses might be easier to access than regional or state-level warehouses."

Page 16, line 1-2

The items highlighted above become important because the paper/data isnt necessarily making the argument that the supply chain interventions reduced stock outs of antihypertensives. More patient were put on treatment, volumes increased but there is no

measurement of increase in forecast accuracy, or the data do not reveal a significant reduction in stock outs across products. Which is understandable given a program was started from scratch. But then the paper talks about some of these items more qualitatively and draws some inferences. If that is the methodological plan, then it depends on a strong logical model of why a particular intervention would lead to an improvement in a specific outcome. Such an approach would then require relating the interventions in this paper to health product supply chain interventions which have been tested rigorously, etc.(e.g. Siedman & Atun 2017, Vledder et al 2018, Leung et al 2016).

We agree with the reviewer. The drugs are one of the five interventions under "India Hypertension Control Initiative" to improve the hypertension treatment and control. We did nor design a separate study for supply chain interventions, hence logic model approach was not used. In the initial planning and implementation phase, lack of drugs were a major challenge. Hence we planned supply chain interventions described in the manuscript. Considering the limited literature on this topic for NCD drugs in context of LMIC, we believe that our experience can offer important insights to researchers and program managers. Our experience can be used to design more structured well designed studies. We have acknowledged the limitation and added in the limitations paragraph of discussion.

Page 22, para 2

In summary I think this is an important area of work. The paper needs to clearly separate where there is a clearly observed change in output/outcome variables (e.g. volume procured, # of patients, protocol choice, etc) and where there isnt a clearly observable difference yet (e.g stock outs). Then for the latter part relate it to literature and logic model. Also, be very clear on what parts are prerequisites e.g. state level procurement governance, presence of reputable private suppliers who can deliver to districts in the state (which may not be the case for some far flung districts in other states), etc.

<u>Author Response</u>

We thank the reviewer for acknowledging the importance of our work. We can only comment on issues which were captured in our results. For instance, we cannot comment on suppliers.

We have modified the conclusions below based on the suggestions.

“We demonstrated the feasibility of strengthening procurement and supply chain management for antihypertensive drugs in a low-resource setting at scale. We were able to document improvement in several important areas, including the adoption of the treatment protocol, increased procurement volumes due to forecasting and collaboration among various stakeholders to match requirements with program scale-up, monthly stock monitoring at the facility level, improved availability of antihypertensive drugs included in the protocol in primary care facilities and minimum 30 days refills for the patients. There were challenges which could not be fully addressed, including timely tendering to ensure uninterrupted rate contracts, multiple suppliers as a risk mitigation strategy, timely placement of purchase ordering and supply schedules and timely availability of transport at the facility level for collecting drugs from the warehouse. Effective practices established in the IHCI can be replicated in other districts in India and in other LMICs to increase the coverage of hypertension treatment. The interventions to further strengthen the governance and adoption of best procurement practices should be sustained to ensure the availability of drugs for NCDs.”
