# Supplementary material for: Improving the availability of antihypertensive drugs in the India Hypertension Control Initiative, India, 2019–2020
Source: PLoS One. 2023 Dec 14;18(12):e0295338. doi: 10.1371/journal.pone.0295338 (PMC10721057; doi:10.1371/journal.pone.0295338)
Supplement: Attachment — Submitted filename: Response to the reviewers comments - plos one.docx [file pone.0295338.s008.docx]

**Improving the availability of antihypertensive drugs in the India Hypertension Control Initiative, India, 2019-2020**

**General Comment:**

Author response:

We are thankful to the Reviewer for their positive comments and putting forward very relevant queries, addressing to which shall help making the paper more robust. Public health supply chains are complex with multiple stakeholders and multi-level decision making. The policies and process framework also differ from state to state. Public health systems in India have limited experience of handling large scale chronic disease control program which requires uninterrupted availability of specific drugs. Under IHCI we planned interventions for each step of the supply chain process, starting from the product selection to dispensing. While the intervention levels are generic, the implementation plan and tools were curated to suit requirements of a scaling-up program.

What evidence does it create about the impact of the interventions carried out and how they impact difference performance objectives. (e.g. stock out rates being one of them).

Uninterrupted availability of drugs is single most important indicator of supply chain system performance. While our study does not have the baseline data to present magnitude of improvement, the evidence is based on three quantitative indicators as described in the methods and results. The indicators include

i.	Quantity of antihypertensive drugs procured for three financial years (2017-18, 2018-19, and 2019-20)

ii.	The proportion of health facilities with drug stock out Out of all implementing facilities, the number of facilities had nil stock of any protocol drug at the end of each quarter from Q2, 2019 to Q1, 2020.

iii.	Drug stock in patient-days at the end of each quarter at the district level from Q2, 2019 to Q1, 2020.

Page 9, section 2.4 (b)

Should other states carry out the full package of interventions described?

States planning to scale up hypertension control programs should adopt a package of interventions including drug-dose specific treatment protocol, forecasting using tools, multi-year rate contracts, a ready reckoner at the facility level and key indicators for monitoring drug availability.

Page 19, first para discussion

While I agree with the authors that choosing a single protocol, forecasting based on patient enrolment vs historical consumption, and basic stock management may be worth expanding to all districts, it is unclear if those interventions will work without good procurement and contracting practices, transparency, and robust governance etc. So how does the choice and prioritization depend on the system, governance, and context in a given state?

We appreciate the reviewer for highlighting the importance of procurement governance in strengthening the supply chain system. We have highlighted some of the issues on page XX. We acknowledge that study did not fully address these issues. This has been added to the discussion. “Robust governance, efficiency and transparency in procurement and contracting practices are essential to improve drug availability. We could not fully document and address these challenges in our study. We need more in-depth studies to explore these challenges and better overall coordination between the program managers and procurement teams to reduce delays.”

Page 21, Para 2

Is there a pre-requisite set of conditions in procurement governance which have to be in place before this can be implemented in a state?

Yes, we agree with the reviewer. The government of India encourages all states to develop autonomous procurement agencies. To implement the interventions, the states should have a department/agency that oversees the procurement process, a well-defined procurement policy to govern supplier selection and contract management, a coordination mechanism between the user department and procurement unit, a risk mitigation plan for supply/quality failures and timely payment mechanism.

Page 21, para 2

**Specific comments**

Some of the supply chain processes of forecasting, procurement, etc are the same for all health products and are not unique to antihypertensives alone. So, would such an intervention work for any drug on the EML if the package of interventions is implemented? it will be useful to bring out more clearly what parts (if any) of the supply chain for antihypertensives are different from essential medicines more broadly?

Author response

Thanks for highlighting this important issue. The interventions such as drug-dose specific treatment protocol, multi-year rate contracts and key indicators for monitoring drug availability at the facility level are relevant for all disease conditions. However, forecasting tools and ready reckoners are useful for hypertension or other chronic diseases where patients need to take drugs on a long-term basis.

Page 20, para 1

There is literature that compares when to use morbidity base forecasts, patient enrolment-based forecasts, historical stock data-based forecasts, etc. A key answered question here is when should a system transition to using consumption-based forecasts? Initially as the program is ramping up it makes sense to use patient enrolment-based forecasts but a few years down as the program achieves equilibrium is there an inflection point where a different forecasting algorithm may be needed?

Author response

We thank the reviewer for this important query. Given the high prevalence and unmet needs, it is reasonable for the public sector to target the enrolment of at least 50 % of the total hypertensive patients. Hence morbidity-based methods are relevant until a large proportion of expected patients are enrolled. We have added this to the discussion. “The morbidity-based estimation method will be useful until the enrolment of a large proportion of patients who are expected to take treatment in the public sector. Once the district has achieved saturation based on utilization of public sector health facilities, the transition to the consumption-based method can be considered based on the assessment.”

Page 21

The paper does not delve into the frequency of replenishment of each health facility. More frequent replenishment/delivery to health facilities reduces the reliance on facility forecasts for long intervals.

Author response:

We thank the reviewer for the opportunity to clarify the long-term forecasting and periodic stock replenishment. Typically, drug requirement forecasting is done annually to prepare a procurement plan and budget. Therefore long-term forecasting is a pre-requisite for ensuring an adequate quantity of drugs can be procured in the year. We have now included the frequency of stock replenishment at health facilities in the results section (table 1, page 12). Details added in the results. “At the facility level, stocks were replenished on a monthly basis. The refill quantity was assessed using ready reckoners (S5 File, S6 File). The monthly cycle helped in overcoming the storage-related challenges at the facility level.”

Page 15, section 3.4 para 2

Similarly, the paper does not delve into the supply chain network design aspects. Is it OK to have state level and district level stores? Should there be more direct distribution from State warehouses to health facilities?

We thank the reviewer for suggesting including supply chain network design aspects in the manuscript. Two of the four states included in the study had district-level stores, while Punjab and Telangana have regional stores from which the drugs are distributed to the health facilities (table 1). As mentioned earlier, the facilities do monthly refills. The pharmacists from health facilities sent an indent, arranged transport, and collected drugs from the primary/district levels stores, usually once a month. Due to transport challenges, it isn't easy to visit regional /state stores, which are far away from facilities. We have added this in the manuscript “Given these constraints, district-level warehouses might be easier to access than regional or state-level warehouses.”

Page 16, line 1-2

The items highlighted above become important because the paper/data isnt necessarily making the argument that the supply chain interventions reduced stock outs of antihypertensives. More patient were put on treatment, volumes increased but there is no measurement of increase in forecast accuracy, or the data do not reveal a significant reduction in stock outs across products. Which is understandable given a program was started from scratch. But then the paper talks about some of these items more qualitatively and draws some inferences. If that is the methodological plan, then it depends on a strong logical model of why a particular intervention would lead to an improvement in a specific outcome. Such an approach would then require relating the interventions in this paper to health product supply chain interventions which have been tested rigorously, etc.(e.g. Siedman & Atun 2017, Vledder et al 2018, Leung et al 2016).

We agree with the reviewer. The drugs are one of the five interventions under “India Hypertension Control Initiative” to improve the hypertension treatment and control. We did nor design a separate study for supply chain interventions, hence logic model approach was not used. In the initial planning and implementation phase, lack of drugs were a major challenge. Hence we planned supply chain interventions described in the manuscript. Considering the limited literature on this topic for NCD drugs in context of LMIC, we believe that our experience can offer important insights to researchers and program managers. Our experience can be used to design more structured well designed studies. We have acknowledged the limitation and added in the limitations paragraph of discussion.

Page 22, para 2

In summary I think this is an important area of work. The paper needs to clearly separate where there is a clearly observed change in output/outcome variables (e.g. volume procured, # of patients, protocol choice, etc) and where there isnt a clearly observable difference yet (e.g stock outs). Then for the latter part relate it to literature and logic model. Also, be very clear on what parts are prerequisites e.g. state level procurement governance, presence of reputable private suppliers who can deliver to districts in the state (which may not be the case for some far flung districts in other states), etc.

Author Response

We thank the reviewer for acknowledging the importance of our work. We can only comment on issues which were captured in our results. For instance, we cannot comment on suppliers.

We have modified the conclusions below based on the suggestions.

“We demonstrated the feasibility of strengthening procurement and supply chain management for antihypertensive drugs in a low-resource setting at scale. We were able to document improvement in several important areas, including the adoption of the treatment protocol, increased procurement volumes due to forecasting and collaboration among various stakeholders to match requirements with program scale-up, monthly stock monitoring at the facility level, improved availability of antihypertensive drugs included in the protocol in primary care facilities and minimum 30 days refills for the patients. There were challenges which could not be fully addressed, including timely tendering to ensure uninterrupted rate contracts, multiple suppliers as a risk mitigation strategy, timely placement of purchase ordering and supply schedules and timely availability of transport at the facility level for collecting drugs from the warehouse. Effective practices established in the IHCI can be replicated in other districts in India and in other LMICs to increase the coverage of hypertension treatment. The interventions to further strengthen the governance and adoption of best procurement practices should be sustained to ensure the availability of drugs for NCDs.”
